# The effects of power ultrasound (24 kHz) on the electrochemical reduction of CO_2_ on polycrystalline copper electrodes

**DOI:** 10.1016/j.ultsonch.2020.105401

**Published:** 2020-12-03

**Authors:** Md Hujjatul Islam, Hamed Mehrabi, Robert H. Coridan, Odne S. Burheim, Jean-Yves Hihn, Bruno.G. Pollet

**Affiliations:** a Hydrogen Energy and Sonochemistry Research Group, Department of Energy and Process Engineering, Norwegian University of Science and Technology (NTNU), Trondheim, Norway; b Microelectronics-Photonics Program, University of Arkansas, Fayetteville, AR, USA; c Department of Chemistry and Biochemistry, University of Arkansas, Fayetteville AR, USA; d UTINAM UMR 6213 CNRS, Université Bourgogne Franche-Comté, Besançon, France

**Keywords:** Sonoelectrochemistry, CO_2_ electrochemical reduction (CO2RR), hydrogen evolution reaction (HER), Methane, Formic acid, Ethanol

## Abstract

The electrochemical CO_2_ reduction reaction (CO2RR) on polycrystalline copper (Cu) electrode was performed in a CO_2_-saturated 0.10 M Na_2_CO_3_ aqueous solution at 278 K in the absence and presence of low-frequency high-power ultrasound (*f* = 24 kHz, *P*_T_ ~ 1.23 kW/dm^3^) in a specially and well-characterized sonoelectrochemical reactor. It was found that in the presence of ultrasound, the cathodic current (*I*_c_) for CO_2_ reduction increased significantly when compared to that in the absence of ultrasound (*silent* conditions). It was observed that ultrasound increased the faradaic efficiency of carbon monoxide (CO), methane (CH_4_) and ethylene (C_2_H_4_) formation and decreased the faradaic efficiency of molecular hydrogen (H_2_). Under ultrasonication, a ca. 40% increase in faradaic efficiency was obtained for methane formation through the CO2RR. In addition, and interestingly, water-soluble CO_2_ reduction products such as formic acid and ethanol were found under ultrasonic conditions whereas under *silent* conditions, these expected electrochemical CO2RR products were absent. It was also found that power ultrasound increases the formation of smaller hydrocarbons through the CO2RR and may initiate new chemical reaction pathways through the sonolytic di-hydrogen splitting yielding other products, and simultaneously reducing the overall molecular hydrogen gas formation.

## Highlights

• Cathodic current (*I_c_*) of CO2RR increased significantly in the presence of ultrasound.
• Ultrasound increased Faradaic efficiency of CO_2_-reduced products such as CO, CH_4_ and C_2_H_4._
• Faradaic efficiency of CH_4_ was increased by ca. 40% in the presence of ultrasound.
• Faradaic efficiency of H_2_ was decreased under ultrasound.
• Ultrasound could initiate new reaction pathways yielding new CO_2_-reduced products.

## 1 Introduction

The conversion of CO_2_ to useful products is of significant value as CO_2_ could, in principle, replace fossil fuels as a feedstock in the chemical industry, enabling a pathway for sustainable chemicals. In this context, the electrochemical reduction of CO_2_ (CO2RR), seen as a clean and controllable energy conversion technology, could be a promising solution to potentially close the “anthropogenic carbon cycle” [1]. This is due to the fact that the CO2RR process converts carbon dioxide into more reduced forms and can generate a wide range of value-added products [1]. Hence, there is a significant interest in the electrochemical CO2RR into hydrocarbon fuels; coupling such a process to renewable electricity could generate carbon–neutral fuels for use in stationary power and transport sectors [1].

The CO2RR is a highly complex reaction with many reaction pathways where the branching ratios are dependent upon a large range of parameters and experimental conditions such as: electrolyte composition, electrolyte pH, electrode material, electrode surface structure, electrode morphology, electrode potential, pressure, temperature, electrochemical cell design and hydrodynamic conditions (e.g. electrolyte or electrode agitation, see later). Numerous reactions proceed simultaneously at the electrode surface, giving rise to a portfolio of different products [2]. For example, the CO2RR leads to major products such as carbon monoxide (CO), formate or methanoate (HCO_2_^−^), formic acid (HCOOH), methane (CH_4_), ethylene (C_2_H_4_) and ethanol (C_2_H_5_OH). The hydrogen evolution reaction (HER), which is widely regarded as a more kinetically facile reaction (in most electrochemical systems), can compete against CO2RR, decreasing the CO2RR selectivity and product yields mainly due to the large activation barrier for forming the CO_2_-radical (*E*^ø^ = –1.98 V *vs.* SHE) [3]. In the CO2RR, the cathodic reaction is usually [4]:

(1) $$\mathrm{xC}O_{2}+nH^{+}+ne^{-}\to \;products\;+yH_{2}O$$

Since the study of Hori and co-workers in 1985 [5], who quantified gaseous and liquid products from the CO2RR, copper (Cu) is still today the only heterogeneous catalyst that exhibits a great affinity towards the generation of valuable hydrocarbons [2], [6]. For further insights on mechanistic pathways for CO2RR on Cu from both an experimental and a theoretical viewpoint, the reader is invited to consult the relevant scientific literature, including one of the latest comprehensive and critical review papers by Nitopi *et al.* entitled “Progress and Perspectives of Electrochemical CO_2_ Reduction on Copper in Aqueous Electrolyte” [4].

However, investigations related to the effect of mass transfer on the CO2RR are still scarce. It has been shown that, for aqueous systems, sufficient supply of CO_2_ to the electrode surface is critical for an efficient CO2RR process. This is usually achieved by mixing efficiently the electrolyte and CO_2_ in order to increase the potential window whereby the CO2RR is governed by intrinsic reaction kinetics. It was also observed that: (i) for this diffusion-limited process, increasing the electrolyte/CO_2_ mixing leads to increased CO2RR rates due to a decrease in the boundary layer thickness at the electrode surface, and (ii) the hydrodynamics have a direct effect on the local pH change at the electrode surface [7], [8], [9], [10].

Power ultrasound (20 kHz–2 MHz) has been successfully employed to enhance many electrochemical systems and to produce useful gases and materials such as hydrogen [11], and nanomaterials for energy production [12]. It is well-known that the coupling of power ultrasound with a specially design electrochemical cell can impart some remarkable advantages such as electrode surface activation, degassing at the electrode surface, electrolyte degassing, disruption of the *Nernst* diffusion layer (reduction in the diffusion layer thickness, *δ*), and enhancement in mass transfer through the electrode double layer [11] which, cannot be achieved by simply rotating the electrode (*rde* – rotating disc electrode) or stirring the solution. Ohta *et al*. have introduced in 2000 for the first-time the use of intense stirring in the form of power ultrasound (26 kHz) on the CO2RR where they witnessed an increase in the faradaic efficiencies of the CO2RR products [13]. This pioneering work is to the best of our knowledge the single experimental study available to date regarding the use of power ultrasound on the CO2RR process. Taking into account the developments and advancements in sonochemistry (and sonoelectrochemistry) during the last decades, this area deserves further investigation. Particularly, the following research questions need to be answered: (i) to what extent does power ultrasound affect the CO2RR process?, (ii) how does intense agitation induced by ultrasound differs from the agitation caused by simple mechanical stirring on the CO2RR? and, (iii) why is the HER depressed under ultrasonic conditions?

This present study highlights the effects of ultrasound on the CO2RR process, with a particular focus on the contribution of agitation due to convection or cavitation by mass transfer quantification. We have also confirmed the depression of hydrogen production as previously observed by Ohta *et al.* ^13^ and have addressed three possible explanations for this phenomenon.

## 2 Experimental methods

**Fig. 1:**
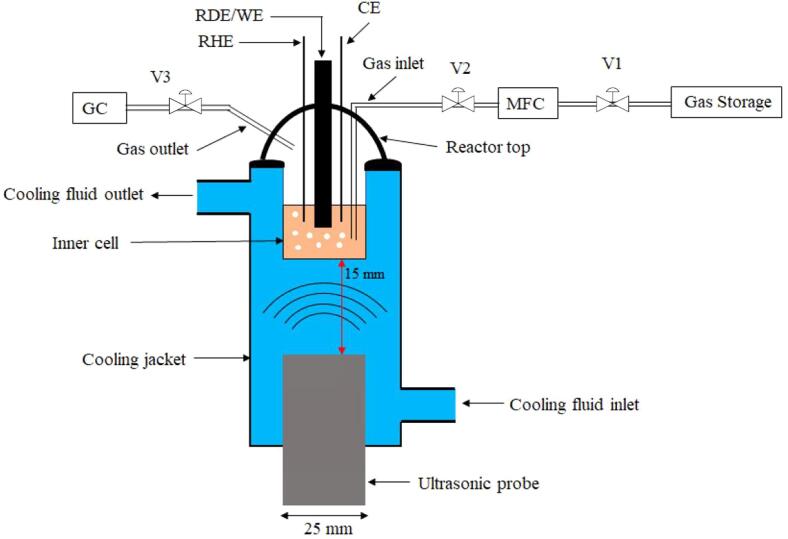
Sonoelectrochemical setup for CO2RR. WE is the Working Electrode, either a RDE (Rotating Disc Electrode) or a wire electrode, RHE is the Reversible Hydrogen Electrode, CE is the Counter Electrode (Pt flag), GC is the Gas Chromatograph, MFC is the Mass Flow Controller, V1, V2 and V3 is the Valve 1, Valve 2 and Valve 3 respectively.

Both mass transfer and CO2RR measurements were performed using a specially designed and well-characterized double jacketed sonoelectrochemical reactor (Besançon cell, Fig. 1) [14]. For the Besançon cell, a double wall reactor, was equipped with a Hielscher Ultrasonics UP400St ultrasonic probe operating at 24 kHz (400 W). The working volume of the inner cell (micro-sonoreactor) was 7 mL. This type of arrangement is known as the “face-on” geometry [15]. In such a configuration, the electroanalyte is not in contact with the ultrasonic probe preventing electrolyte contamination by the damage of the ultrasonic (US) probe as well as electrical issues (the US probe may act as an additional electrode if not grounded properly). The cooling liquid was circulated through the cooling jacket which also acted as a coupling media for the propagation of the ultrasonic energy from the cooling liquid to the reaction media. A mixture of water and monoethylene glycol (MEG) was used as cooling fluid which allowed controlled temperature operations. The microreactor was equipped with a working electrode (WE), a counter electrode (CE), a reference electrode (RE), a gas inlet, a gas outlet and a temperature thermocouple.

For all (sono)electrochemical experiments, a lab fabricated Reversible Hydrogen Electrode (RHE) and Pt foil (0.64 cm^2^, 99.99% pure, Goodfellow Cambridge Ltd) was used as the RE and the CE respectively. The working electrodes (WE) were either a polycrystalline Pt disc (Rotating Disc Electrode - RDE, ∅ = ~3 mm, Metrohm Autolab – for mass transfer experiments), a polycrystalline Cu disc (Rotating Disc Electrode - RDE, ∅ = ~5 mm, Metrohm Autolab – for CO2RR experiments) or a polycrystalline Cu wire (*L* = ~21 mm, ∅ = ~0.95 mm, Goodfellow Cambridge Ltd – for CO2RR experiments). The WE Pt RDE and CE Pt flag electrodes were polished to mirror finish using alumina suspension and immersing them in 25% H_2_SO_4_ solution for 10 mins. The electrodes were rinsed with ultrapure water (18.2 MΩ.cm) and dried before placing into the sonoelectrochemical reactor. A BioLogic, SP-150 potentiostat and an Autolab Rotating Disc Electrode (RDE) from Metrohm were used.

## 3 Equivalent mass transfer measurements

For mass-transfer measurements, a Pt RDE was used as the working electrode (WE) immersed in an equimolar quasi-reversible redox couple of 5 × 10^-3^ mol/L Fe^2+^/Fe^3+^. K_4_Fe(CN)_6_·3H_2_O (CAS: 14459–95-1) and K_3_Fe(CN)_6_ (CAS: 13746–66-2) were purchased from Alfa Aesar and used as Fe^2+^ and Fe^3+^ respectively in 0.2 mol/L Na_2_SO_4_ (CAS: 7757–82-6, purchased from Sigma-Aldrich) background electrolyte solution. Linear Sweep voltammograms (LSV) were recorded under steady-state conditions at a scan rate of 2 mV/s.

**Fig. 2:**
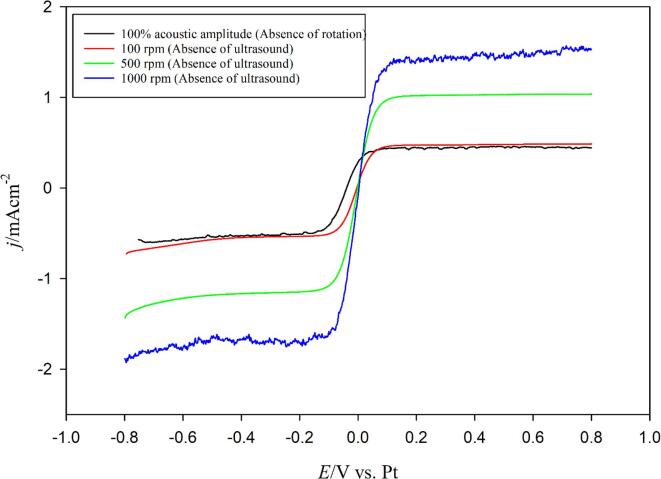
Linear sweep voltammograms (LSV) for equimolar quasi-reversible redox couple of 0.005 mol/L Fe^2+^/Fe^3+^ in 0.2 mol/L Na_2_SO_4_ at a scan rate 2 mV/s.

At first, the LSVs (Fig. 2) were performed on a Pt RDE at 100% acoustic amplitude (24 kHz) and the *k*_d_ values from the LSVs (in the potential window of *E* = –0.8 V to + 0.8 V *vs.* RHE) were calculated. LSVs were also performed under rotating conditions (in the absence of ultrasound) and rotation speeds (*ω*) were adjusted to find the equivalent *k*_d_ at the equivalent rotation speed (*ω*_eq_) corresponding to the 100% acoustic amplitude. It was found that the *k*_d_ value (1.06 × 10^-5^ m/s) for 100% ultrasonic amplitude nearly corresponded to the *k*_d_ value (1.11 × 10^-5^ m/s) of 100 rpm rotation speed.

**Fig. 3:**
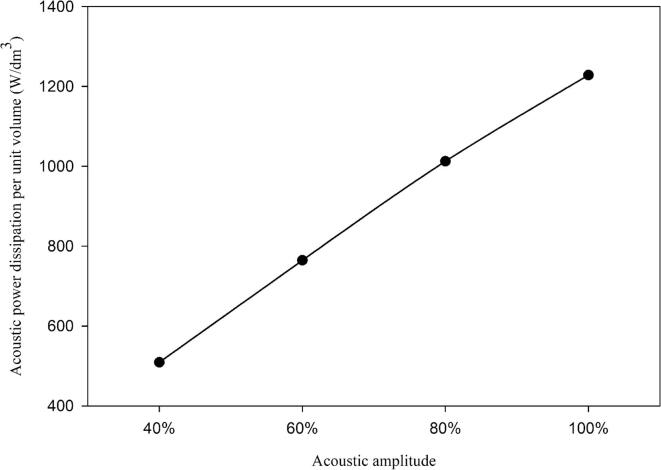
The transmitted acoustic power dissipated per unit volume at various ultrasonic amplitudes.

In addition, the transmitted acoustic power (*P*_T_) was measured at various ultrasonic amplitudes using the method presented by Mason *et al.* [16] and Contamine *et al.* [17]*.* In this method, a thermocouple was placed in the inner reactor containing ultrapure water (7 mL). The circulation of the cooling/coupling fluid was stopped. The temperature equalized with the reactor sample and the calorimetry experiments were performed thereafter. The temperature increase, due to the conversion of mechanical energy into heat, was recorded every second by using a National Instruments thermocouple controlled by a LabView software. Herein, the acoustic powers are quoted as W/dm^3^. Fig. 3 shows the transmitted acoustic power dissipated per unit volume at different ultrasonic amplitudes.

## 4 CO2RR experiments

For the CO2RR measurements, either a polycrystalline Cu RDE or a polycrystalline Cu wire (99.99% pure, Goodfellow Cambridge Ltd) electrodes were used as the working electrode (WE) immersed in a CO_2_ saturated 0.1 mol/L Na_2_CO_3_ electrolyte (Na_2_CO_3_·10H_2_O, purity: 99.999% trace metal basis, CAS: 6132–02-1, Sigma Aldrich). Before each experiment, the Cu RDE tip and Cu wire electrodes were activated by anodic polarization in 14.7 mol/L H_3_PO_4_ (CAS: 7664–38-2, Sigma Aldrich) at + 0.5 A for 100 s which ensure a stable oxide layer onto the copper surface. 0.1 mol/L Na_2_CO_3_ was used as electrolyte which was saturated by bubbling CO_2_ at a rate of 250 mL/s by using a mass flow controller (Alicat Scientific) for 30 mins ensuring CO_2_ saturation of the solution and removal of dissolved oxygen (DO) simultaneously. The solubility of CO_2_ was also measured at different temperatures (5, 15 and 30 °C) using an InPro 5000i sensor manufactured by Mettler Toledo in both pure water and 0.1 mol/L Na_2_CO_3_ for comparison purposes. The pH of the saturated solution, prior, during and after the experiments, was measured using a pH meter (Multiparameter Meter edge®, Hanna Instruments). It was found that at 5 °C, the solubility of CO_2_ reached maxima of 2,380 mg/L for pure water and 2,590 mg/L for Na_2_CO_3_. On the other hand, the final pH values of the CO_2_ saturated pure water and 0.1 mol/L Na_2_CO_3_ were found to be 3.8 and 6.8 respectively. In this study, all CO2RR experiments were performed in CO_2_ saturated 0.1 mol/L Na_2_CO_3_ solutions regulated at 5 °C.

Linear sweep voltammograms (LSV) and cyclic voltammograms (CV) experiments of CO_2_ saturated in 0.1 mol/L Na_2_CO_3_ electrolytes at Cu RDE and Cu wire electrodes were performed from the rest potential to –1.4 V *vs.* RHE, in the absence and presence of ultrasound (at 100% acoustic amplitude only) at scan rates of 1, 5 and 50 mV/s. For comparison purposes, CVs (50 mV/s) of Cu electrodes immersed in N_2_ saturated 0.1 mol/L Na_2_CO_3_ electrolytes were also performed. In addition, LSV experiments were carried out using a Cu RDE (in the absence of ultrasound) at the equivalent rotation speed (*ω*_eq_) (found in mass-transfer experiments at 100% acoustic power) in order to investigate the effects of ultrasound [18].

Finally, chronoamperometry (CA) experiments were performed at –1.4 V *vs.* RHE for 15 mins in the absence and presence of ultrasound (24 kHz, 100% acoustic amplitude). A Cu wire and Pt flag electrodes were used as the WE and the CE respectively. The charges (*Q*) from the CA curves were determined using the EC-Lab software. Faradaic efficiencies (FE) were calculated using equation (1):

(1) $$\mathrm{FE}=\frac{n\times z\times F}{Q}\times 100\%$$

where *n* is the number of moles of gaseous products in the gas phase, *z* is the number of electrons transferred in the CO2RR to produce the product, *F* is the Faraday constant (96,485.3C/mol) and *Q* is the charge in C.

During all CA experiments, the sonoelectrochemical reactor was completely gas tight. A 100 µl sample of the headspace atmosphere was collected immediately after each CA experiment using a Vici Series A-2 gas syringe. The sample was injected into a gas chromatograph (GC; Model 8610C, SRI Instruments) for product analysis using both thermal conductivity detector (TCD) and flame ionization detector (FID) as detectors. The GC used a 1.8 m Hayesep-D column with argon (Ar) for a carrier gas. The GC was equipped with a TCD for H_2_ detection and a FID for detecting volatile organics such as CO, CH_4_, and C_2_H_4_. The analysis of the products and faradaic efficiencies were computed from the GC data based on calibration experiments that used small molecule calibrant standards (Restek Corp.).

The liquid products were collected and analyzed by nuclear magnetic resonance spectroscopy (^1^H NMR) using a Bruker 500 MHz liquid-phase NMR. The cell solution from each experiment was mixed in a 9:1 mass ratio with D_2_O (Sigma-Aldrich). Dimethyl sulfoxide (DMSO; Sigma-Aldrich) was used as an internal standard due to its single ^1^H peak at a chemical shift of 2.7 ppm. Data was collected using solvent suppression to reduce the ^1^H signal from the water at roughly 5 ppm. Chemical shifts for all of the products of interest here were outside of the region of artefacts caused by the solvent suppression. To confirm that any products found in the NMR experiments were derived from CO2RR and not from contamination of the buffer solution or the purge gas, a sample of the head space (GC) and solution (NMR) before the experiments were analyzed.

## 5 Results and discussion

### 5.1 Cyclic voltammetry (CV) and linear sweep voltammetry (LSV) studies

**Fig. 4:**
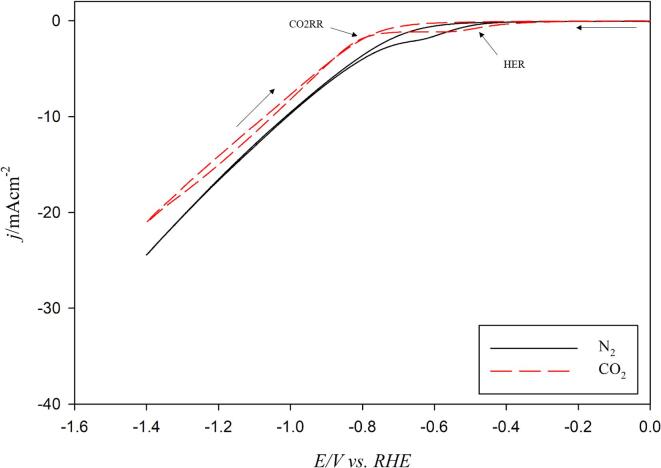
Cyclic voltammograms (CV) for a polycrystalline Cu wire immersed in a N_2_ saturated 0.1 mol/L Na_2_CO_3_ and a CO_2_ saturated (2,590 mg/L) 0.1 mol/L Na_2_CO_3_ electrolyte at 50 mV/s in the absence of ultrasound.

Fig. 4 shows two cyclic voltammograms (CVs) in the range of [–1.40 V < *E* < 0.00 V *vs.* RHE] for a polycrystalline Cu wire immersed in a N_2_ saturated 0.1 mol/L Na_2_CO_3_ (pH = 11.4) and a CO_2_ saturated (2,590 mg/L) 0.1 mol/L Na_2_CO_3_ (pH = 6.8) at a scan rate of 50 mV/s in the absence of ultrasound and at 278 K. In the presence of N_2_, the CV shows a typical electrochemical behaviour for copper in a mild carbonate solution as already observed in the literature [19] i.e. the presence of a reduction current at around −0.3 V *vs.* RHE (onset potential), corresponding to the hydrogen evolution reaction (HER) which is, in our conditions, diffusion limited [19]. In the presence of CO_2,_ the HER diffusion-limited plateau is more pronounced, with a lower current value within a larger potential window [–0.6 - –0.8 V *vs.* RHE]. The equilibrium potentials of CO_2_ reduction and HER reduction are in the same potential range in aqueous electrolytes. At ca. –0.8 V *vs.* RHE, a current is observed which is usually attributed to the CO2RR [19] from either the dissolved CO_2_ or the bicarbonate anions. This reaction is clearly in competition with the HER, and should yield CO, CH_4_ and other hydrocarbons [19], [20]. At high cathodic potentials (*E* < –1.35 V *vs.* RHE), either proton or water reduction also occurs producing more hydrogen than CO2RR products. At higher pH where the H^+^ concentration is low, water reduction is also expected to dominate over H^+^ reduction [8].

It was previously observed that the HER and the CO2RR processes, deplete H^+^ or produce OH^–^ and a ΔpH can establish at the electrode surface, yielding several competing effects on these reactions due to a complicated interplay between mass transport, buffer equilibria, and bulk pH [8]. Some debate exists as to whether the HER proceeds via the H^+^ or water reduction, and whether high local pH is beneficial or detrimental toward the CO2RR [4]. According to Ooka *et al.* [21], thermodynamically, HER should not depend on pH (on the RHE scale), and in theory, any Brønsted acid could act as a H^+^ donor. The same workers showed [21] that the HER occurs primarily via water reduction under CO2RR conditions and it may also be possible that the electrolyte buffer could act as a H^+^ donor, depending on its pKa value, concentration, mass transport and reactant availability at the electrode surface. Some other studies have shown that: (i) increasing the local pH promotes the CO2RR over the HER, mainly due to the decreasing overpotential for the formation of C_2+_ products, and (ii) local pH shifts the acid − base reactions equilibria toward (bi)carbonates, which may reduce CO_2_ concentration at the electrode surface, in turn promoting the HER instead [22], [23].

**Fig. 5:**
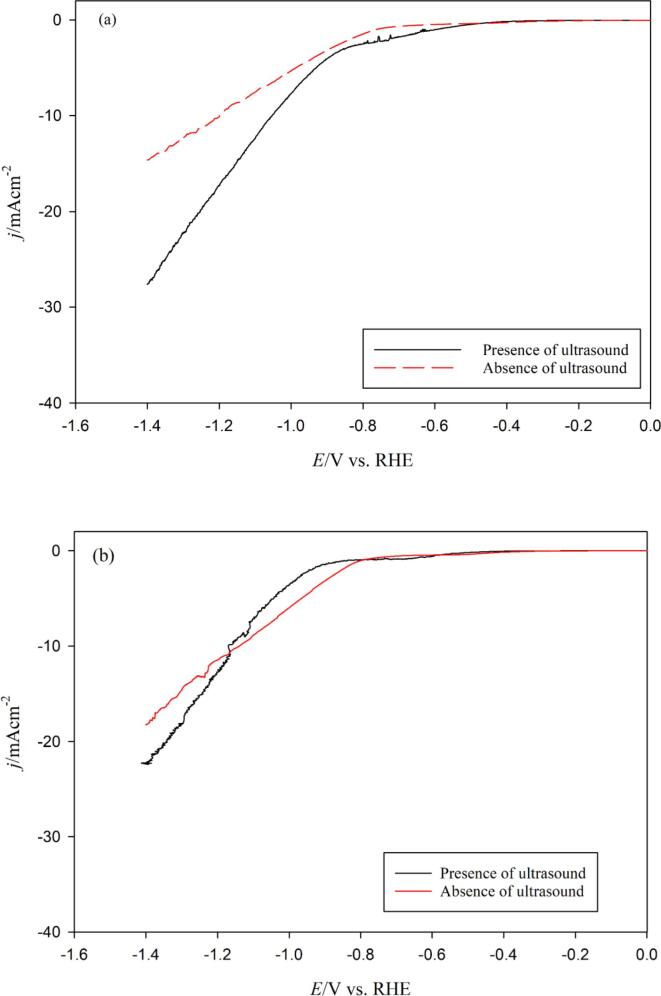
Linear sweep voltammograms (LSV) for a polycrystalline Cu wire immersed in a CO_2_ saturated (2,590 mg/L) 0.1 mol/L Na_2_CO_3_ electrolyte at (a) 1 mV/s and (b) 5 mV/s and at 278 K in the absence and presence of ultrasound (100% acoustic amplitude, 24 kHz).

As this system shows limitations with mass transfer, LSVs were recorded in CO_2_ saturated solutions in the presence of ultrasound to investigate the effect of high stirring on the CO2RR and HER (Fig. 5). It is well-known that power ultrasound enhances mass transfer of electroactive species from the bulk solution to the electrode surface. This elevated mass transfer occurs due to the sono-physical effects caused by acoustic streaming, high velocity liquid jets induced by cavitation bubble implosion, and efficient bulk electrolyte stirring [11], [24], [25]. Under *silent* conditions and CO_2_ saturation, decreasing the scan rate to “near steady-state” i.e. 1 mV/s (Fig. 5(a)) leads to a significant decrease in the HER current, but both the HER and the CO2RR onset potentials remains in the same range of magnitude (*E*_onset,HER_ = –0.520 V *vs.* RHE and *E*_onset,CO2RR_ = –880 V *vs.* RHE) than those observed at a scan rate of 50 mV/s (Fig. 4, *E*_onset,HER_ = –0.420 V *vs.* RHE and *E*_onset,CO2RR_ = –0.810 V *vs.* RHE). At 1 mV/s scan rate and in the presence of ultrasound, the current corresponding to the hydrogen evolution is greatly improved due to the enhanced mass transfer and an important shift toward more negative potentials is observed for the CO2RR, i.e. a Δ*E*_onset,CO2RR_ of ca. –0.20 V. A possible explanation lies in the enhancement of proton (and hydroxide ions) consumption from the HER and CO2RR under ultrasonic conditions, in turn leading to an increase in a local pH at the vicinity of the electrode surface. This finding is in good agreement with that observed in the literature, in which at higher pHs, the HER becomes dominant due to mass transfer limitations of CO_2_ [8]. Another explanation is that, under ultrasound, the (bi)carbonate species balance is modified with possible precipitation of hydroxides which may reduce the electrode surface access or at least a lack of availability of dissolved CO_2_. It was shown that, for the CO_2_/bicarbonate system, CO_2_ acts both as a reactant and a buffer, thus a pH increase near the cathode surface may cause the dissolved CO_2_ concentration to deviate (and even decrease) from that in the bulk electrolyte [8]. Moreover, for a scan rate of 1 mV/s, the cathodic current density above –1.0 V *vs.* RHE is higher in the presence of ultrasound than in the absence of ultrasound.

Increasing the scan rate to 5 mV/s (Fig. 5(b)) yields a reduction in the HER plateau due to kinetic reasons, with the HER onset potential values being similar for both *silent* and ultrasonic conditions. This observation may indicate that protons (and OH^–^) consumption is reduced by a great amount, the presence of (bi)carbonates has lesser effects and interface cleanliness of the electrode occurs. Moreover, it was observed that CO2RR shifts towards more cathodic potentials at higher scan rate because of the poisoning of surface sites by adsorbed intermediates associated with the reduction of CO_2_ to CO. The intermediate products take finite time to accumulate on the cathode surface for further reduction enabling more cathodic potential to be reached[8]. This is quantitatively measurable with the shift of CO2RR onset potentials i.e. a potential shift of a Δ*E*_onset,CO2RR_ of ca. –0.120 V (*E*_onset,CO2RR,Silent_ = –0.830 V *vs.* RHE and *E*_onset,CO2RR,US_ = –0.950 V *vs.* RHE). At a 50 mV/s scan rate, the kinetic is fast that little and even no changes in the electrode/electrolyte interface polarization can take place and both LSVs recorded in presence or absence of ultrasound present exactly the same behavior with identical onset potentials for HER and CO2RR (not shown here).

The presence of a limiting current plateau in the HER onset potential region is worthy of a complementary discussion. It is well-known that the composition and concentration of anions and cations (and their electrostatic interactions), buffer capacity, pH, and availability of H^+^ donors affect the HER [26] and CO2RR [6], [8], [19]. Mukouyama *et al.* [27] postulated that a decrease of the HER current might be due to the decrease in the electromigration transport of protons from the bulk solution to the electrode surface in the presence of cations such as sodium (Na^+^). Their proposed explanation is that, the presence of cations either as Na^+^ or as K^+^ affects (mainly reduces) the H^+^ transport electromigration to the electrode surface. Murata and Hori demonstrated that the CO_2_ reduction selectivity on polycrystalline Cu was strongly influenced by cation size, with larger cations increasing the selectivity toward the formation of C_2+_ species and decreasing the selectivity for the HER [28].

**Fig. 6:**
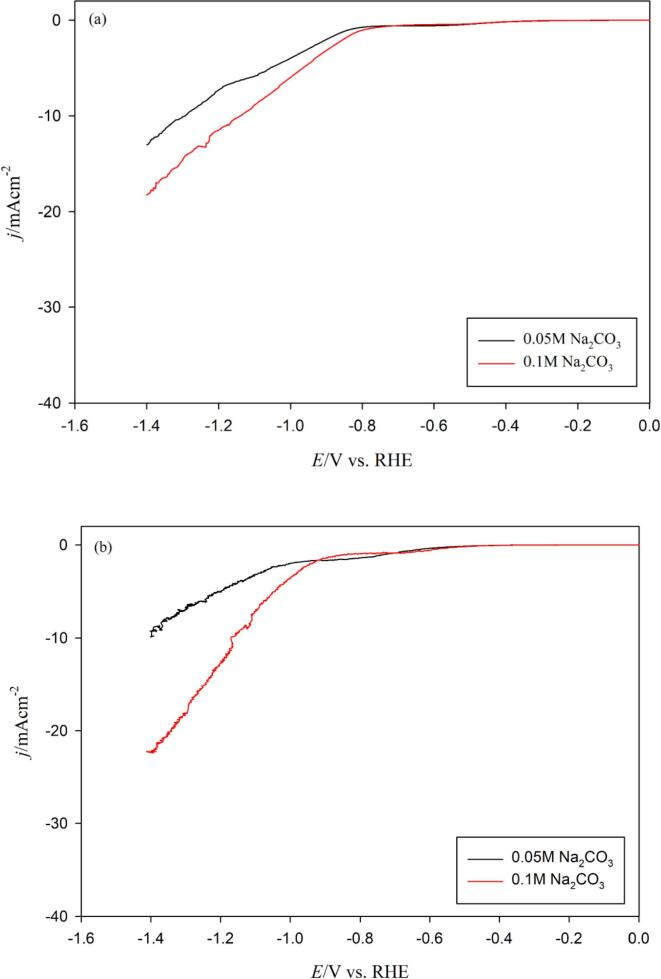
Linear sweep voltammograms (LSV) for a polycrystalline Cu wire immersed in a CO_2_ saturated (2,590 mg/L) 0.1 mol/L and 0.05 mol/L Na_2_CO_3_ electrolyte at 5 mV/s and at 278 K in the (a) absence of ultrasound and (b) presence of ultrasound (100% acoustic amplitude, 24 kHz).

Thus, the effect of cation (Na^+^) concentration (0.05 M Na_2_CO_3_ and 0.10 M Na_2_CO_3_) on the HER and the CO2RR in the absence and presence of ultrasound was studied by recording LSVs at a scan rate of 5 mV/s in the range of [0.0 V *vs.* RHE - –1.4 V *vs.* RHE] as shown in Fig. 6. From Fig. 6(a) (*silent* conditions), no obvious Na^+^ concentration effect on the HER process can be observed, although a slight decrease in the limiting-diffusion current can be seen, a finding which is less evident than that observed by Mukouyama *et al.* [27], possibly due to the difference in scan rate employed. However, it can be clearly observed that: (i) the CO2RR onset potential shifts to more positive potentials, and (ii) current densities over –1.0 V *vs.* RHE are higher with increasing Na^+^ concentration, possibly due to a lower amount of carbonate.

In presence of ultrasound, the effect of Na^+^ concentration is much more pronounced with a significant increase in the HER diffusion-limited plateau at the lowest concentration (Fig. 6(b)). At 0.05 M Na_2_CO_3_, ultrasound not only affects the electromigration transport of protons from the bulk solution to the electrode surface in the presence of Na^+^, but also increases the HER current at the plateau, and shifts significantly the CO2RR onset potential toward more negative values (Δ*E*_onset,CO2RR_ ≈ –0.110 V). Once again, favoring the HER may lead to a local pH increase, which is detrimental to the CO_2_/bicarbonate balance and thus the CO2RR.

**Fig. 7:**
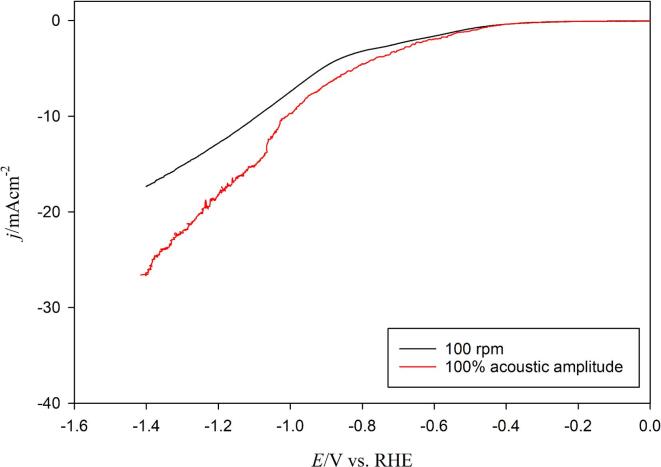
Linear sweep voltammograms (LSV) at the equivalent rotation speed (no ultrasound, *ω*_eq_ = 100 rpm) and at 100% acoustic amplitude (24 kHz) for polycrystalline Cu disc electrodes in a CO_2_ saturated (2,590 mg/L) 0.1 mol/L Na_2_CO_3_ electrolyte at 50 mV/s and at 278 K.

These findings are in good agreement with those observed by Surendranath *et al*. [29], [30] and Goyal *et al.* [20] who showed that: (i) the CO2RR rates are either not affected by agitation (in the form of electrode rotation) or decreased with increasing rotation speed, and (ii) the HER is increasing with increasing RDE rotation rate. Nevertheless, in the case of ultrasonic conditions, a distinction should be made between mass transfer effect and more specific ones such as, electrode improvements due to surface modification, or chemical transformations induced by radical formation (sonolysis) close to the electrode surface. To this purpose, LSVs were recorded (shown in Fig. 7) under ultrasonic (100% amplitude, 24 kHz) and *silent* conditions i.e. at the equivalent rotation (100 rpm) using a RDE, in other words at the corresponding rotating speed which gave a *k*_d_ equivalent to the one obtained under 100% acoustic amplitude under ultrasound conditions (see Fig. 2). It is important to note that to enable the comparison with a RDE, the working electrode geometry was changed from a Cu wire to a Cu disc (same material supplier), reducing the accessibility and modifying slightly the “global” electrochemical behaviour. In these conditions and at the same equivalent *k*_d_, the effects induced by ultrasound are much more prominent than by a simple agitation caused by the rotation of the RDE Cu tip. It can be observed that the cathodic current density improved significantly above the HER potential window, but also remained always higher under sonication, especially after the start of the CO2RR. This is particularly interesting because for large scale set-up, mass transfer might be mandatory to ensure a good regeneration of reactants from the bulk electrolyte to the electrode surface. In the case of ultrasound, mass transfer is present, but it is also associated to a combination of several additional effects allowing a clear CO2RR improvement.

### 5.2 Chronoamperometry, NMR and GC studies

From the LSV study and in the absence of ultrasound, the onset potential for CO2RR at 5 °C was found to be around –0.8 V *vs.* RHE. Since methane is produced in the higher negative potential range (and it is the main target product for this study), a working electrode potential of –1.4 V *vs.* RHE was applied for 15 min for the chronoamperometry (CA) experiments in the absence and presence of ultrasound (24 kHz, 100% acoustic amplitude) at 5 °C (since CO_2_ solubility is maximum at that temperature).

**Fig. 8:**
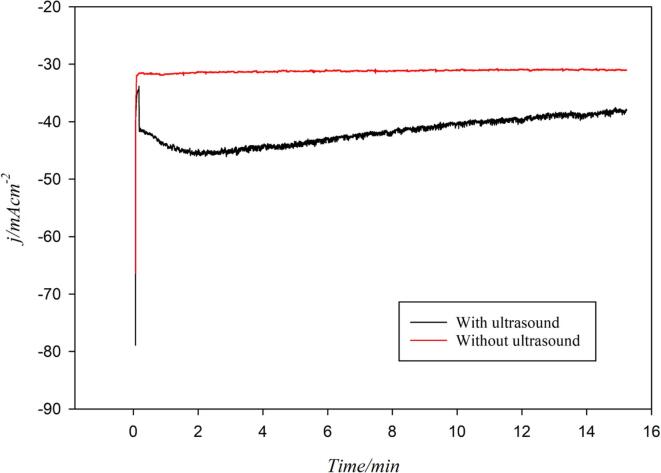
Chronoamperometry (CA) study of a CO_2_ saturated 0.1 mol/L Na_2_CO_3_ electrolyte at 5 °C and at –1.4 V *vs.* RHE on a polycrystalline Cu wire electrode in the absence and presence of ultrasound (24 kHz, 100% acoustic amplitude).

The CA curves in the absence and presence of ultrasound (24 kHz, 100%) are shown in Fig. 8. In the absence of ultrasound, the cathodic current density was found to be –30 mA/cm^2^ at an applied cathode potential of –1.4 V *vs.* RHE and under ultrasonication, the overall cathodic current was on average 1.3-fold higher than that obtained under *silent* conditions. The initial increase of the cathodic current is due to ultrasound bringing about large quantity of dissolved CO_2_ from the bulk solution to the electrode surface, in turn yielding a thinning of the *Nernst* diffusion layer (*δ*). After ca. 2 min of sonication, the cathodic current peaked at –45 mA/cm^2^ and then stabilized at around –40 mA/cm^2^ for the remaining 13 min, possibly due to solution degasification induced by ultrasound and the establishment of a CO_2_ equilibrium between the gas phase and the liquid phase. However, since all CA experiments were performed in a gas tight reactor, a portion of the degassed CO_2_ could have been released and accommodated in the gas phase of the reactor vessel resulting in a slight pressure increase. In contrast, in the absence of ultrasound, the system was not disturbed and provided a constant current all the way from the beginning until the end of the experiment.

**Fig. 9:**
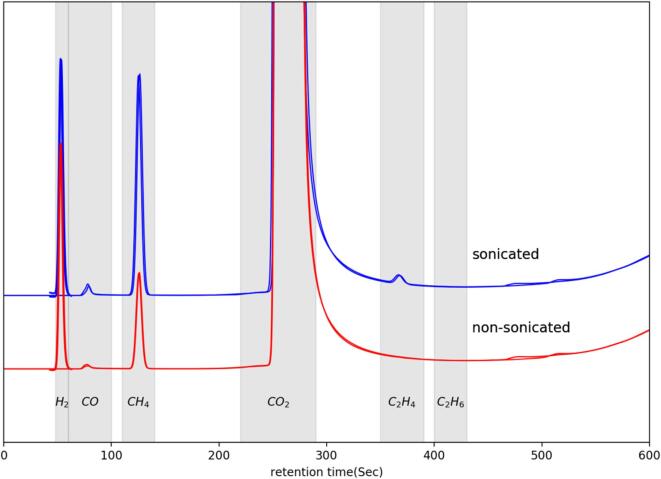
Gas chromatogram (GC) of the gaseous products from the chronoamperometry (CA) study of a CO_2_ saturated 0.1 mol/L Na_2_CO_3_ solution at 5 °C and at –1.4 V *vs.* RHE on polycrystalline Cu wire electrode in the absence and presence of ultrasound (24 kHz, 100% acoustic amplitude).

**Fig. 10:**
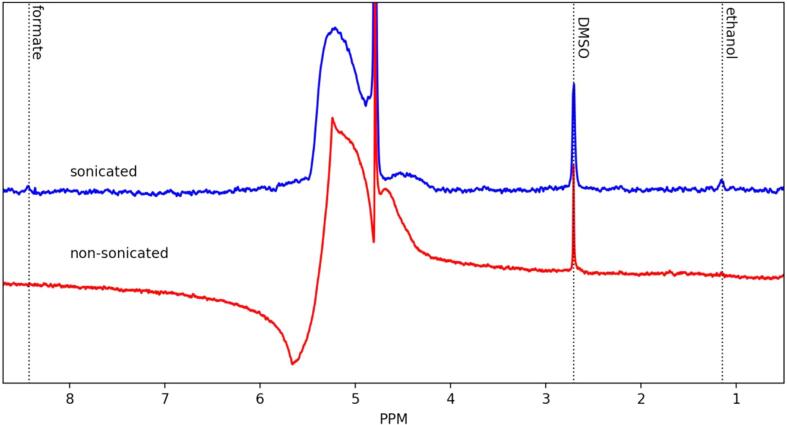
NMR of the liquid products from the chronoamperometry (CA) study of a CO_2_ saturated 0.1 mol/L Na_2_CO_3_ solution at 5 °C and at –1.4 V *vs*. RHE on a polycrystalline Cu wire electrode in the absence and presence of ultrasound (24 kHz, 100% acoustic amplitude).

After 15 min, gaseous samples from the headspace of the reactor were collected and injected into the GC for analysis. The gas chromatograms obtained from the GC for *silent* and sonicated samples are presented in Fig. 9. The formation of CO and various hydrocarbons through the CO2RR and the production of H_2_ through proton and water reduction was observed both in the absence and presence of ultrasound. Under *silent* conditions, the CO2RR products were found to be mainly CH_4_ with a small amount of CO. However, in the presence of ultrasound, formation of C_2_H_4_ was also observed. Based on the NMR analysis of the liquid products (Fig. 10), it was found that ultrasound also produced water soluble CO_2_ reduction products such as formic acid and ethanol. In the absence of ultrasound, no water-soluble CO_2_ reduction products were found which is not in agreement with previous findings observed in the literature [6]. In fact, formic acid and ethanol are two of the primary water-soluble products of CO2RR on Cu electrode. The CO2RR in these experiments were performed in a single cell where both working (Cu) and counter (Pt) electrodes were immersed together in the same electrolyte. Carbon monoxide and formic acid have a high affinity to be adsorbed on platinum [20]. In our conditions, it could be thus assumed that carbon monoxide, formic acid and ethanol were also formed under *silent* conditions, and that most of the formic acid and ethanol had been oxidized back to CO_2_ including a portion of the CO. Moreover, a small amount of CO formation was also observed under *silent* conditions which could have escaped into the gas phase before being oxidized by the platinum counter electrode. On the other hand, in the presence of ultrasound, the adsorption of these products at the platinum counter electrode could have been severely disturbed, hindering further oxidation to CO_2_. Another possibility could be that the initiation of a new CO2RR electrochemical reaction pathways was triggered by ultrasound. For example, Ohta *et al*. [13] proposed a new electrochemical CO2RR reaction mechanism, catalyzed by both H• and OH• radicals formed by ultrasonication resulting in the formation of CH_4_, CO and HCOOH. Based on the chemical dosimetry study, the formation of a small amount of OH• radicals were observed (results not presented here). Therefore, the formation of HCOOH and CH_3_CH_2_OH in the presence of ultrasound could be due to: (i) the inability to be oxidized by the platinum counter electrode or/and (ii) the new electrochemical CO2RR reaction pathways influenced by ultrasonication.

The faradaic efficiencies (FE) of the CO_2_ reduced gaseous products were calculated and are presented in Table 1. For methane formation, the faradaic efficiency was found to be 11% in the absence of ultrasound. However, in the presence of ultrasound, the faradaic efficiency for methane formation increased from 11% to 19% i.e. a ca. 50% increase in FE was observed in presence of ultrasound. Moreover, in the presence of ultrasound, the faradaic efficiency was increased for all CO_2_ reduction products. Interestingly, on the other hand, the faradaic efficiency of H_2_ formation decreased in the presence of ultrasound i.e. the faradaic efficiencies of H_2_ was 88% and 68% in the absence and presence ultrasound respectively. A similar finding was also observed by Ohta *et al.* [13] where faradaic efficiency of hydrogen production was decreased under ultrasonic irradiation. The specific reason for this suppression of the hydrogen production under ultrasonication is still unknown. However, three possible explanations could be addressed in order to shed some light on the findings:

(i) It is possible that ultrasound promotes the CO2RR over the HER, mainly due to the decreasing overpotential for the formation of C_2+_ products such as C_2_H_4_ and C_2_H_5_OH [8].
(ii) Ultrasonication of the aqueous electrolyte solutions produces OH**^•^** radicals (via sonolysis) [11], and a fraction of the produced hydrogen (dissolved) might be scavenged by the OH**^•^** radicals according to equation (2) as proposed by Gutierrez *et al.* [31].

(2) $$OH^{\cdot }+H_{2}\to H_{2}O+H^{\cdot }$$

**Table 1:** Faradaic efficiency (FE) analysis from the chronoamperometry (CA) study of a CO_2_ saturated 0.1 mol/L Na_2_CO_3_ electrolyte at 5 °C and at –1.4 V *vs.* RHE on a polycrystalline Cu wire electrode in the absence and presence of ultrasound (24 kHz, 100% acoustic amplitude).

Conditions	Time(min)	Charge, *Q*(C**)**	Overall Faradaic Efficiency (FE)(%)				FE ratio		Total FE(%)
			H_2_	CO	CH_4_	C_2_H_4_	CO/H_2_	CH_4_/H_2_	
*Silent*	15	18.72	88.51	0.14	11.09	0.15	0.0016	0.13	99.89
Ultrasound (24 kHz, 1.23 kW/dm^3^)	15	25.12	68.31	0.22	19.00	0.70	0.0032	0.28	88.23

As proposed by Ohta *et al*. [13] the produced hydrogen radical (H**^•^**) could then take part in the sono-CO2RR reaction mechanism pathway presented in equations (7–9).

For CO2RR and in the absence of ultrasound, the below mechanism has been proposed [13]:

(3) $$CO_{2}\overset{e^{-}}{\to }{\;}^{\cdot }CO_{2}^{-}\overset{CO_{2}+e^{-}}{\to }CO+CO_{3}^{2-}$$

(4) $${\;}^{\cdot }CO_{2}\overset{H^{+}+e^{-}}{\to }CO+OH^{-}\overset{4H^{+}+4e^{-}}{\to }{\;}^{\cdot }CH_{2}+H_{2}O\overset{2H^{+}+2e^{-}}{\to }CH_{4}$$

(5) $${\;}^{\cdot }CO_{2}^{-}\overset{H^{+}+e^{-}}{\to }HCOO^{-}$$

(6) $${\;}^{\cdot }CH_{2}+{\;}^{\cdot }CH_{2}\to C_{2}H_{4}$$

For CO2RR and in the presence of ultrasound, a sono-CO2RR mechanism has also been proposed [13]:

(7) $${\;}^{\cdot }CH_{2}+H^{\cdot }\to {\;}^{\cdot }CH_{3}\overset{H^{\cdot }}{\to }CH_{4}$$

(8) $${\;}^{\cdot }CO_{2}^{-}+H^{\cdot }\to HCO_{2}^{-}\to CO+OH^{-}$$

(9) $$\mathrm{CO}+H^{\cdot }\to {\;}^{\cdot }COH\overset{{\;}^{\cdot }OH}{\to }HCOOH$$

Therefore, it is possible that the electrochemically produced molecular hydrogen might have been consumed through the radical induced sono-CO2RR reaction pathways giving rise to elevated amount of CO2RR products such as CH_4_, C_2_H_4_, CO, HCOOH. The increase of faradaic efficiency for CH_4_ in the presence of ultrasound could be due to the combination of both classical CO2RR and sono-CO2RR taking place simultaneously.

(iii) It is also possible that the electrochemically produced molecular hydrogen might have been trapped inside the cavitation bubble generated by ultrasonication. It is well-known that, upon collapse, cavitation bubbles produce enormous amount of energy with temperature and pressure of ca. 5,000 K and 2,000 atm, respectively [11]; and under these extreme conditions, homolytic fission of molecular H_2_ may occur according to equation (10).

(10) $$H_{2}\overset{\mathrm{Ultrasonication}}{\to }H^{∙}+H^{∙}$$

H^•^ could then take part in the sono-CO2RR reactions producing hydrocarbons. Therefore, in these conditions, the HER reaction is not suppressed under sonication, although, a fraction of molecular hydrogen could be either scavenged by the OH**^•^** radicals or “sonolyzed” (eq. (10)) due to cavitation bubble collapse.

## 6 Conclusions

This study focused on the effects of power ultrasound on the electrochemical CO_2_ reduction process. In the presence of ultrasound, it was observed that: (i) the CO2RR onset potential shifts to more positive potentials (Δ*E* = +0.170 V), and (ii) current densities over –1.0 V *vs*. RHE are higher with increasing Na^+^ (as Na_2_CO_3_) concentration (by ~ 2-fold), possibly due to a lower amount of carbonate. By increasing Na^+^ concentration, it was found that ultrasound not only affects the electromigration transport of protons from the bulk solution to the electrode surface, but also increases the HER current in the plateau region and shifts significantly the CO2RR onset potential to more negative values. This could possibly create a local pH increase, which might be detrimental to the CO_2_/bicarbonate balance and thus the CO2RR.

In addition, equivalent mass transfer study has revealed that even at equivalent *k_d_*, the mass transfer caused by ultrasonication in CO2RR is much higher (by ~ 1.5-fold) than mechanical stirring (RDE). From the chronoamperometry study and by analyzing the gaseous and liquid products, it was found that ultrasound increases the faradaic efficiency of methane by ca. 2-fold. In some cases, ultrasound could initiate radical induced new electrochemical CO2RR pathways giving rise to new products such as C_2_H_4_, HCOOH, and CH_3_CH_2_OH.

As observed in the pioneering work by Ohta *et al.* [13], that in the presence of ultrasound the faradaic efficiency of hydrogen formation was decreased. From our quantification and analyses, it could be assumed that hydrogen formation (through HER and H2ORR) appears not to be depressed. The produced hydrogen could be either scavenged by OH**^•^** formed by ultrasonication or could be sonolyzed into H**^•^,** which possibly might take part in the new sono-CO2RR reaction mechanism producing higher quantities of hydrocarbons.

## CRediT authorship contribution statement

**Md Hujjatul Islam:** Conceptualization, Data curation, Formal analysis, Investigation, Methodology, Project administration, Software, Validation, Visualization, Writing - original draft. **Hamed Mehrabi:** Conceptualization, Data curation, Formal analysis, Investigation, Methodology, Software, Writing - review & editing. **Robert H. Coridan:** Conceptualization, Data curation, Formal analysis, Investigation, Methodology, Supervision, Software, Writing - review & editing. **Odne S. Burheim:** Funding acquisition. **Jean-Yves Hihn:** Conceptualization, Data curation, Formal analysis, Investigation, Methodology, Project administration, Resources, Software, Supervision, Validation, Visualization, Writing - review & editing. **Bruno.G. Pollet:** Conceptualization, Data curation, Formal analysis, Funding acquisition, Investigation, Methodology, Project administration, Resources, Software, Supervision, Validation, Visualization, Writing - review & editing.

## Declaration of Competing Interest

The authors declare that they have no known competing financial interests or personal relationships that could have appeared to influence the work reported in this paper.
